# Supplementary material for: Identification of a Partial and Selective TRPV1 Agonist CPIPC for Alleviation of Inflammatory Pain
Source: Molecules. 2022 Aug 25;27(17):5428. doi: 10.3390/molecules27175428 (PMC9457966; doi:10.3390/molecules27175428)
Supplement: Supplementary file 1 [file molecules-27-05428-s001.zip › molecules-1862857-supplementary.pdf]

Supplementary Materials

Identification of a Partial and Selective TRPV1 Agonist CPIPC for Alleviation of Inflammatory Pain

Liying Dong [1,†], Qiqi Zhou [2,†], Qianqian Liang [3,†], Zhen Qiao [3], Yani Liu [1,4,*] , Liming Shao [3,*] and Kewei Wang [1,4,*]

1. Departments of Pharmacology, School of Pharmacy, Qingdao University Medical College, Qingdao 266073, China
2. Department of Pharmacology, Qilu Medical University, Zibo 255300, China
3. Department of Medicinal Chemistry, School of Pharmacy, Fudan University, No. 826 Zhangheng Road, Shanghai 201203, China
4. Institute of Innovative Drug Discovery, Qingdao University Medical College, 38 Dengzhou Road, Qingdao 266021, China

* Correspondence: liuyani@qde.edu.cn (Y.L.); limingshao@fudan.edu.cn (L.S.); wangkw@qdu.edu.cn (K.W.)
[†] These authors contributed equally to this work.

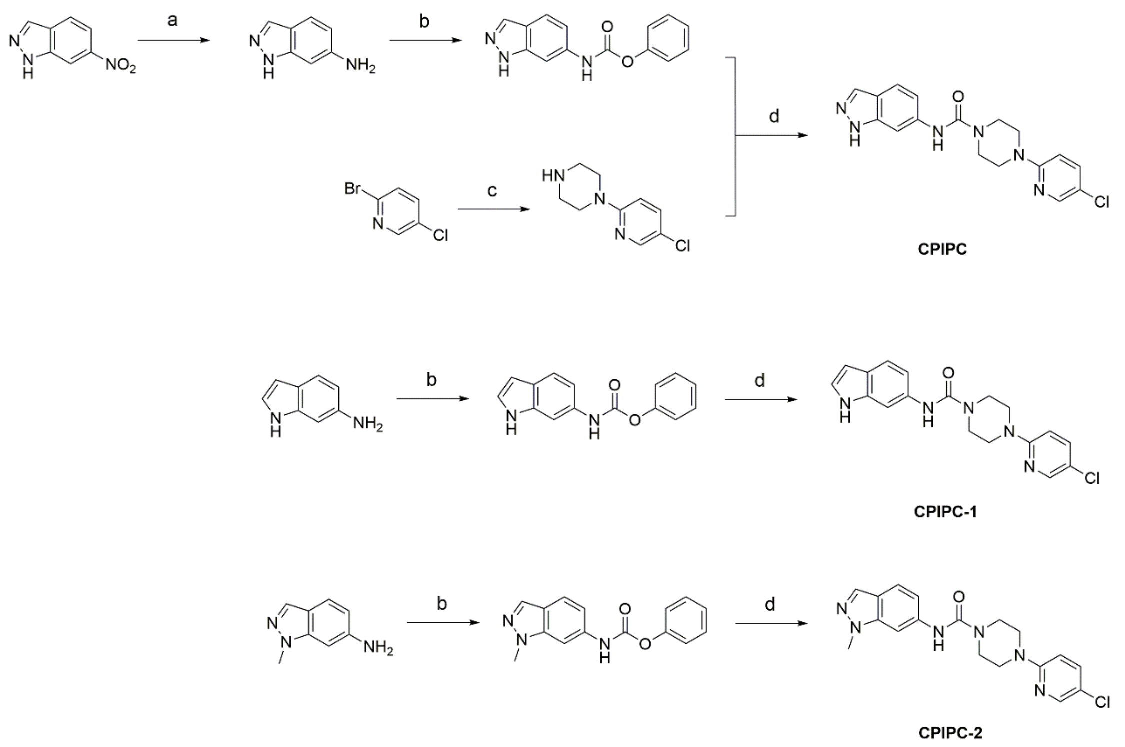

Scheme S1. Synthetic pathway of **CPIPC**, **CPIPC-1** and **CPIPC-2**. Reagents and conditions: (a) H_2, Pd/C, MeOH, rt; (b) PhOCOCl, pyridine, THF, 0 °C → rt; (c) piperazine, *n*-BuOH, 120 °C; (d) *N*-substituted piperazine, Et_3N, DMF, 50 °C.

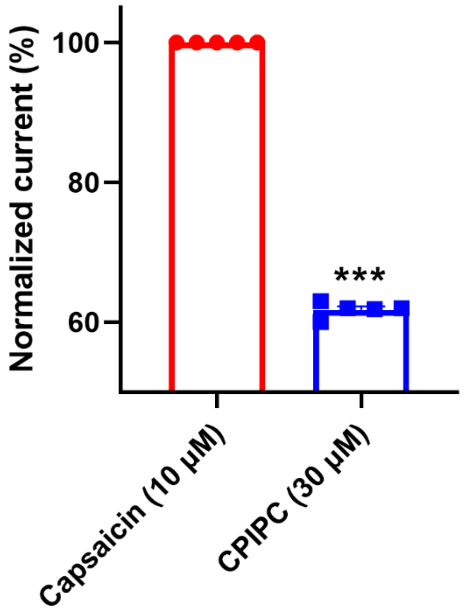

Figure S1. The comparison of *h*TRPV1 current in response to capsaicin (10 μM) and CPIPC (30 μM) (n = 3). *** $p < 0.001$, Paired *t*-test.

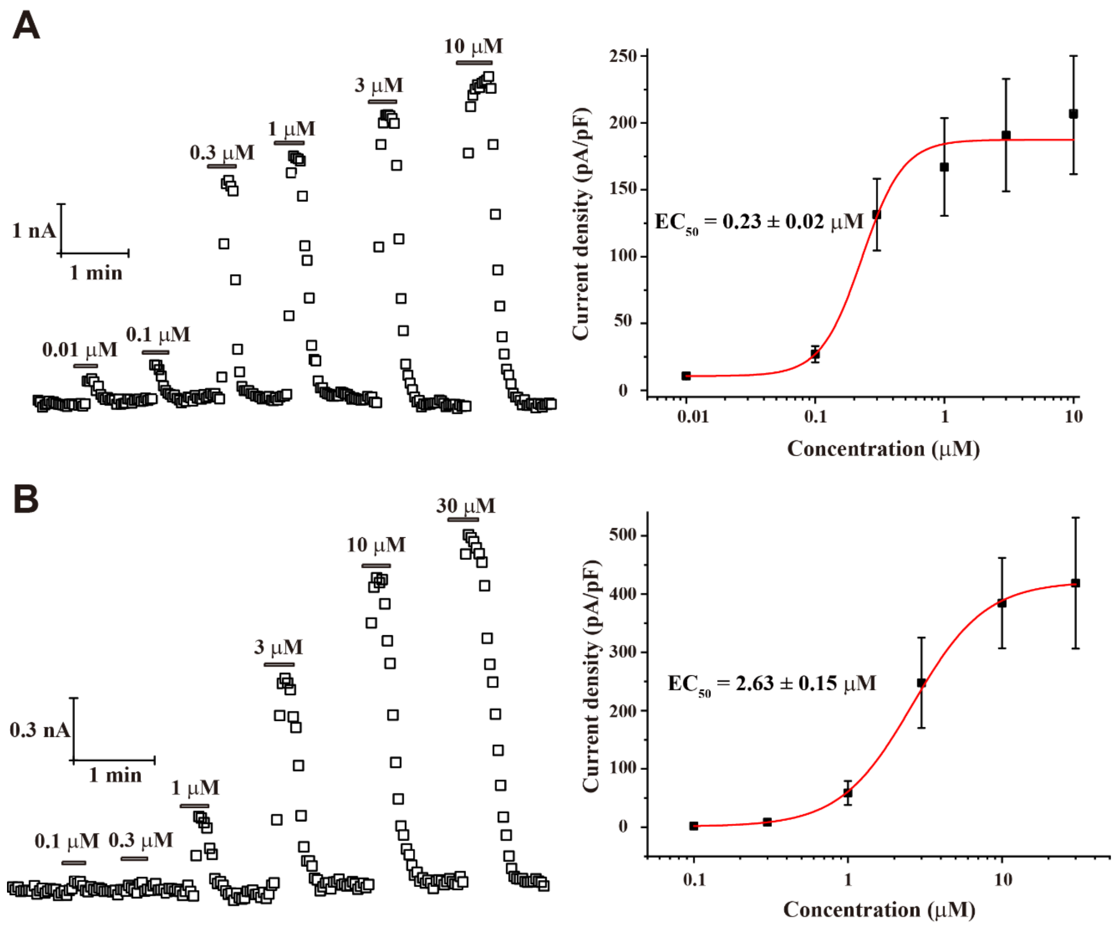

Figure S2. Concentration-dependent activation of TRPV1 currents by capsaicin in HEK293 cells expressing TRPV1 channels with or without **CPIPC**-induced desensitization. (**A**) Left: Capsaicin activated TRPV1 channel currents in response to +100 mV; Right: curve fitting analysis of capsaicin-mediated activation of TRPV1 currents with an EC_{50} of 0.23 ± 0.02 μM (n = 5). (**B**) Left: Concentration-dependent activation of TRPV1 currents by capsaicin in response to +100 mV after **CPIPC**-induced desensitization; Right: Curve fitting analysis of concentration-dependent activation of TRPV1 by capsaicin with an EC_{50} of 2.63 ± 0.15 μM (n = 6).

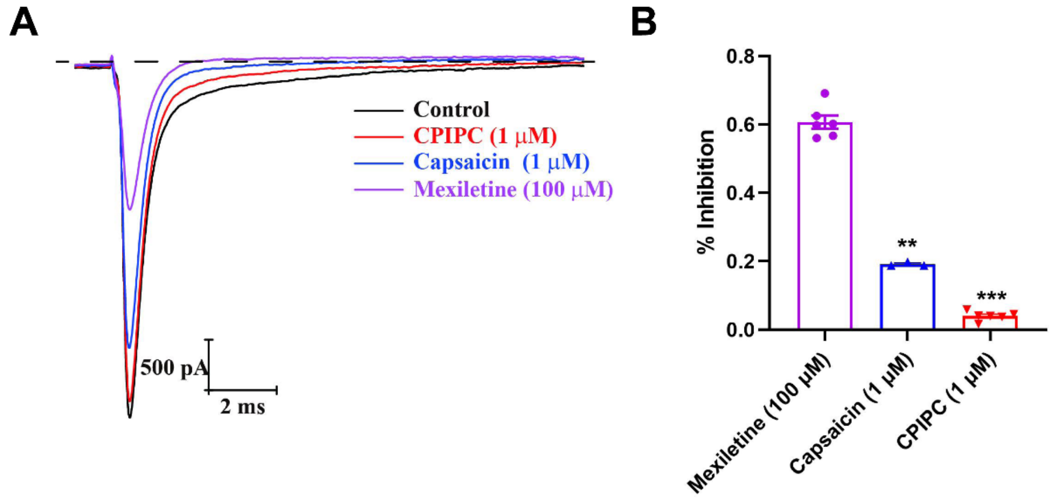

Figure S3. Lack of Nav1.7 inhibition by **CPIPC** in whole-cell patch-clamp recording (n = 3–6). (**A**) Representative Nav1.7 currents elicited by depolarization to 0 mV with holding potential of −130 mV. The dashed line represents zero-current level. (**B**) A summary for Nav1.7 current inhibition by capsaicin (1 μM), **CPIPC** (1 μM) and mexiletine (100 μM). Statistical difference between the Mexiletine and **CPIPC** or capsaicin is indicated as ** $p < 0.01$, *** $p < 0.001$ by one-way ANOVA and *post hoc* Bonferroni's test.

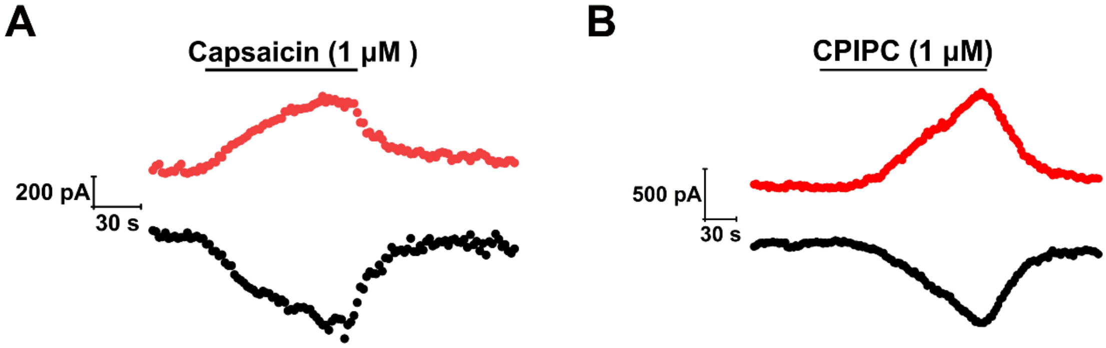

Figure S4. The effect of capsaicin and **CPIPC** on TRRV1 R557A mutant expressed in HEK293 cells. Current traces of R557A TRPV1 mutant in response to 1 μM capsaicin (**A**) and 1 μM **CPIPC** (**B**) recorded at +100 mV (red) and −100 mV (black) in the whole-cell clamp patch.

NMR data obtained on MercuryPlus 400 MHz and Bruker AV-600 MHz spectrometers with DMSO-d_6 as solvent).

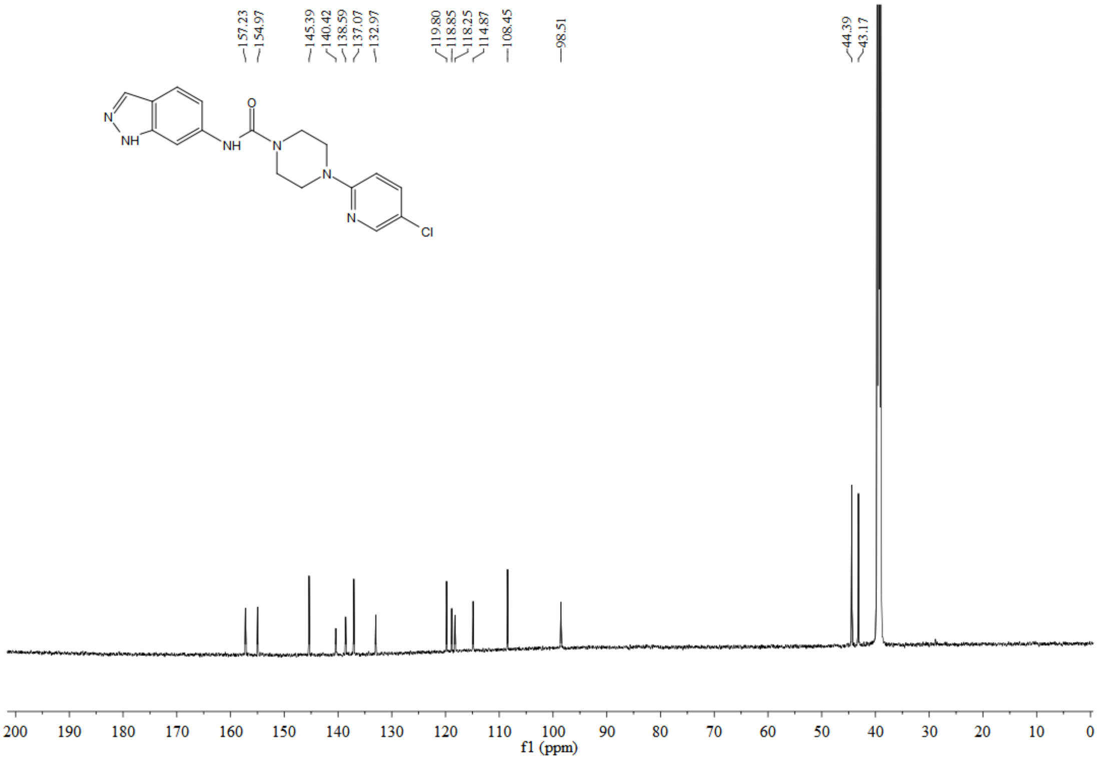

Figure S5. ^{13}C NMR of **CPIPC.**

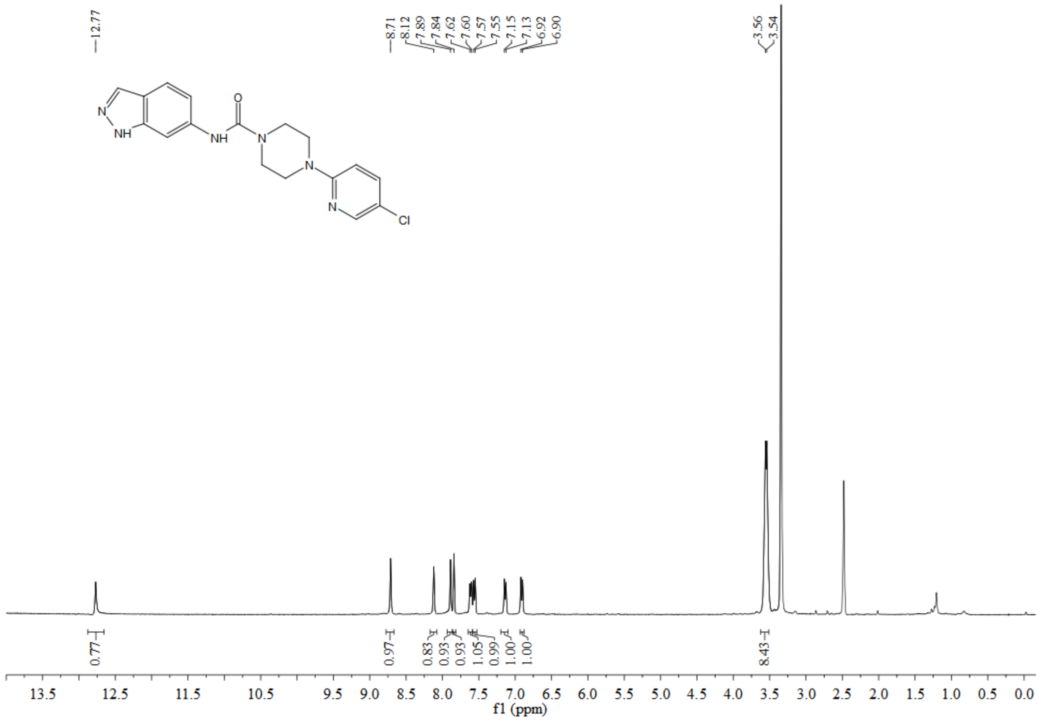

Figure S6. ^{1}H NMR of **CPIPC.**

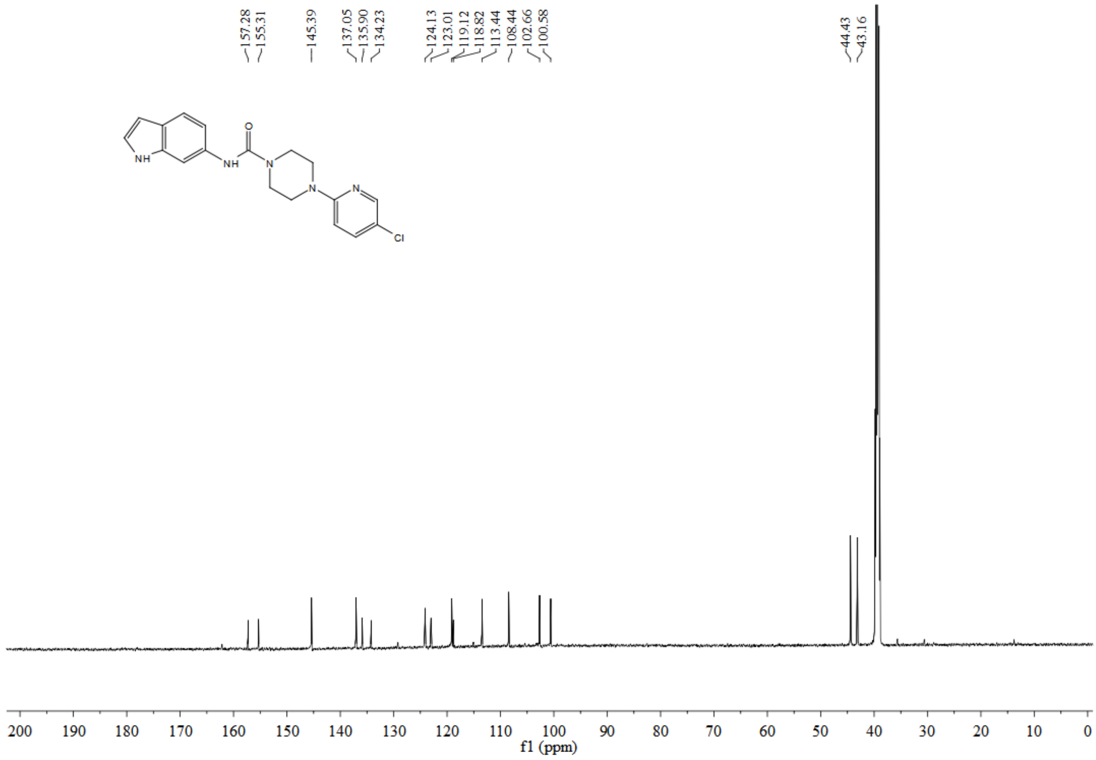

Figure S7. ^{13}C NMR of **CPIPC-1**.

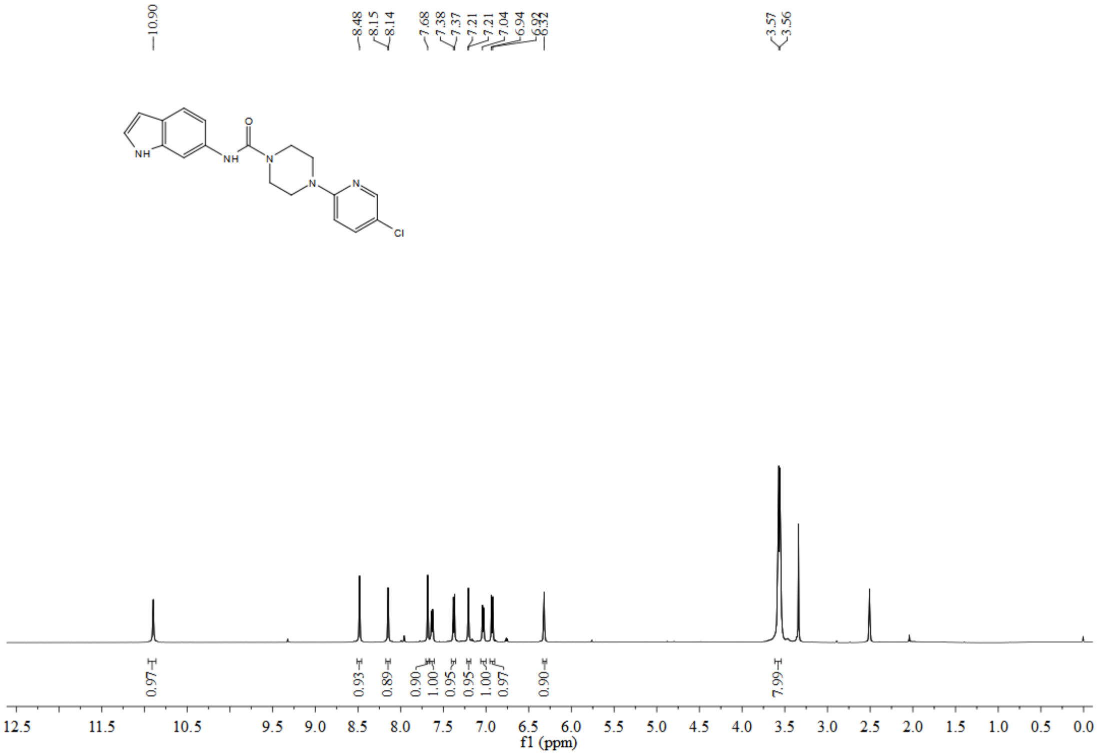

Figure S8. ^{1}H NMR of **CPIPC-1.**

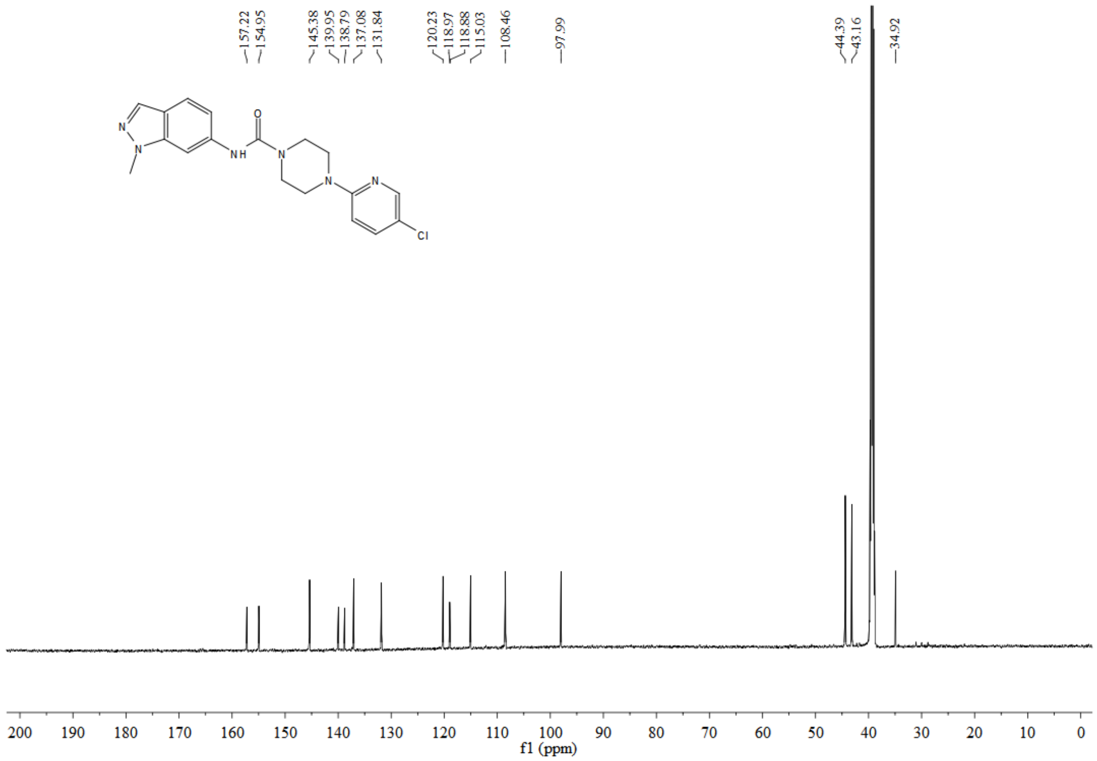

Figure S9. ^{13}C NMR of **CPIPC-2.**

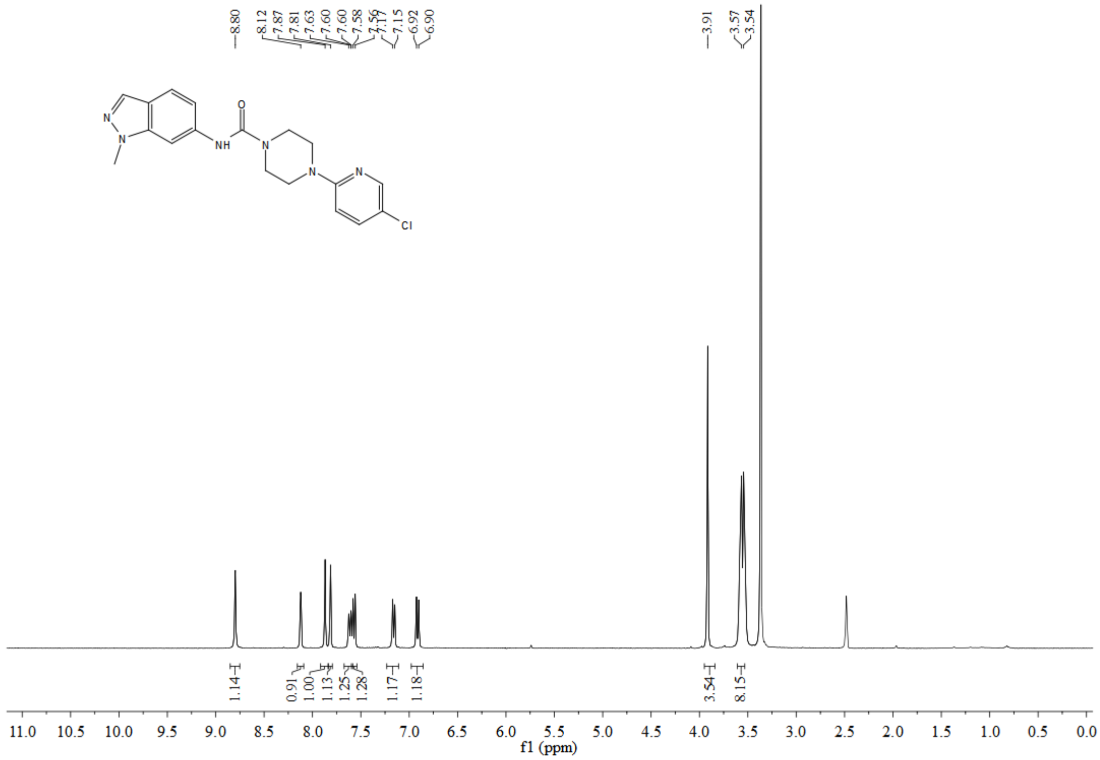

Figure S10. ^{1}H NMR of **CPIPC-2.**
